# EC4, a truncation of soluble N-cadherin, reduces vascular smooth muscle cell apoptosis and markers of atherosclerotic plaque instability

**DOI:** 10.1038/mtm.2014.4

**Published:** 2014-03-26

**Authors:** Cressida A Lyon, Jason L Johnson, Stephen White, Graciela B Sala-Newby, Sarah J George

**Affiliations:** 1 Bristol Heart Institute, Bristol Royal Infirmary, Bristol, UK

**Keywords:** apoptosis, atherosclerosis, cell adhesion molecules, smooth muscle

## Abstract

Atherosclerotic plaque instability is precipitated by vascular smooth muscle cell apoptosis in the fibrous cap, weakening it and leading to plaque rupture. We previously showed that reducing smooth muscle cell apoptosis with soluble N-cadherin (SNC) increased features of plaque stability. We have now identified the active site of SNC and examined whether a truncated form containing this site retains the antiapoptotic effect. SNC was mutated to prevent interaction with N-cadherin or fibroblast growth factor receptor (FGFR). Interaction with FGFR in the extracellular (EC) 4 domain of SNC was essential for the antiapoptotic effect. Therefore, we made a truncated form consisting of the EC4 domain. EC4 significantly reduced smooth muscle cell, macrophage, and endothelial cell apoptosis *in vitro* by ~70%, similar to SNC. Elevation of plasma levels of EC4 in male apolipoprotein E–deficient mice with existing atherosclerosis significantly reduced apoptosis in brachiocephalic artery plaques by ~50%. EC4 reduced plaque size and the incidence of buried fibrous layers and the macrophage:smooth muscle cell ratio (surrogate markers of plaque instability). Interaction of EC4 with FGFR induced potent antiapoptotic signaling *in vitro* and *in vivo*. EC4 modulates atherosclerosis in mice demonstrating its therapeutic potential for retarding plaque size and instability.

## Introduction

A major contributor to atherosclerotic plaque instability and rupture is vascular smooth muscle cell (VSMC) apoptosis. Although VSMC apoptosis is rarely observed in normal blood vessels, in human unstable atherosclerotic plaques, increased apoptosis rates are detected.^1^ Apoptotic VSMCs are located in the protective fibrous cap^2^ and are associated with thinning and loss of fibrous cap integrity.^3^ Mouse models have provided direct evidence that VSMC apoptosis causes an increase in plaque instability.^4–6^ Apoptosis of other cell types, including macrophages, is also observed as lesions develop. Accordingly, reducing apoptosis, and in particular, VSMC apoptosis has been considered as a potential and novel therapeutic strategy to prevent atherosclerotic plaque rupture.

Cadherins are homophilic cell–cell adhesion molecules and are involved in prosurvival signaling in various cell types, including VSMCs. Expression of cadherins is cell type specific, VSMCs express N-cadherin, and overexpression of N-cadherin significantly increase VSMC survival.^4,7^ Soluble N-cadherin (SNC), which consists of the extracellular domain, acts as a mimetic to the full-length molecule and also increases VSMC survival. Importantly, SNC was conjugated to the Fc domain to increase half-life *in vivo* and to ease purification for *in vitro* and *in vivo* studies (SNC-Fc).We have previously shown that SNC-Fc activated the fibroblast growth factor receptor, resulting in activation of the prosurvival phosphoinositide 3 kinase/Akt/phospho-Bad pathway.^8^ SNC-Fc also reduced apoptosis in atherosclerotic plaques and increased features of atherosclerotic plaque stability in apolipoprotein E–deficient mice,^8^ suggesting that SNC-Fc may have therapeutic potential for retarding plaque instability.

A soluble molecule has obvious advantages therapeutically; however, at ~120 kDa, SNC-Fc is a rather large therapeutic molecule, therefore, we aimed to identify the active antiapoptotic site and evaluate whether a smaller molecule containing this site retained the antiapoptotic and plaque stabilizing properties. SNC consists of five extracellular (EC) domains: EC1–EC5. SNC has two binding domains: the homophilic N-cadherin binding domain (HAV/INPISGQ) in EC1 and the FGFR binding domain (INPDVGQ) in EC4.^9^Our previous work suggests that SNC-Fc can activate the FGFR, as well as phosphoinositide 3 kinase and Akt signaling.^8^ However, it was unclear whether (i) SNC-Fc could interact with the FGFR directly; (ii) the interaction of SNC-Fc with N-cadherin on the cell surface was required for FGFR activation; or (iii) the interaction with N-cadherin caused additional activation of phosphoinositide 3 kinase and Akt.^10–12^ To investigate this further, we have mutated the two binding sites to prevent the interaction with either N-cadherin or FGFR.

In this study, we have characterized mutated versions of SNC-Fc to determine the active antiapoptotic site and subsequently truncated the molecule accordingly. We have then investigated whether the truncated form, EC4-Fc, retains prosurvival effects both *in vitro* and *in vivo* and has the potential for repressing plaque instability.

## Results

### Mutation and truncation of SNC

**Figure 1:**
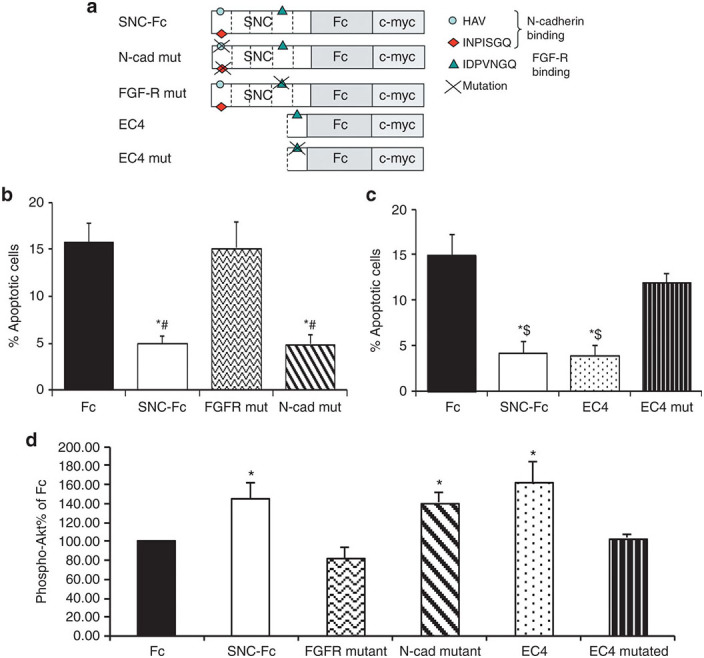
**The effect of SNC-Fc mutation and truncation on vascular smooth muscle cell (VSMC) apoptosis.** (**a**) Diagrammatic representation of the SNC-Fc molecule to show the locations of the fibroblast growth factor receptor (FGFR) and N-cadherin binding sites, the mutations to prevent binding, and truncation of SNC-Fc to EC4-Fc. (**b**,**c**) The percentage of apoptotic VSMCs (assessed by cleaved caspase-3 immunocytochemistry) 24 hours after induction of apoptosis with Fas-L and treatment with the purified proteins (mean ± SEM, *n* = 3, *P < 0.05 versus Fc, ^#^*P* < 0.05 versus FGFR mutated, ^$^*P* < 0.05 versus EC4 mutant). (**d**) EC4 activated Akt signaling to the same extent as SNC-Fc. Analysis of phospho-Akt immunocytochemistry following 24-hour treatment with the purified proteins (mean ± SEM, *n* = 4, *P < 0.05 versus Fc, FGFR mutated and EC4 mutant). EC, extracellular; SNC, soluble N-cadherin.

To determine which part of the SNC molecule was responsible for its antiapoptotic effect, we mutated the two active sites present in the molecule (Figure 1a). SNC can bind to N-cadherin via an interaction between an HAV binding motif in the first extracellular domain (EC1) and with INPISGQ in the EC1 domain of an adjacent molecule. To prevent this interaction, both of these motifs were mutated in SNC. SNC can also interact with the FGFR through INPDVGQ in the EC4 domain; therefore, this motif was mutated to prevent this interaction.

Interestingly, we found that when the N-cadherin binding sites were mutated (N-cad mut), this form of SNC-Fc still reduced VSMC apoptosis (Figure 1b). In contrast, when the FGFR binding site was mutated (FGFR mut), this form of SNC-Fc could no longer reduce Fas-L–induced VSMC apoptosis (Figure 1b). This suggested that the interaction of the FGFR with the EC4 domain of SNC-Fc was essential for the reduction in VSMC apoptosis. Therefore, we truncated SNC-Fc (~120 kDa) to EC4-Fc (~50 kDa). Accordingly, EC4-Fc reduced VSMC apoptosis to an extent similar to that of the full SNC-Fc molecule (Figure 1c). However, when the FGFR binding site within EC4 was mutated (EC4 mut), EC4 no longer reduced apoptosis (Figure 1c).

### EC4-Fc activated the FGFR and increased prosurvival phospho-Akt

We have previously shown that SNC-Fc reduced apoptosis by activating the FGFR, resulting in activation of the downstream prosurvival phosphoinositide 3 kinase/Akt pathway.^8^ Immunocytochemistry for phospho-Akt (a prosurvival factor) demonstrated that EC4-Fc increased phospho-Akt to an extent similar to that of SNC-Fc (Figure 1d), suggesting that it is functioning through the same pathway.

### EC4-Fc reduced macrophage and endothelial apoptosis

**Figure 2:**
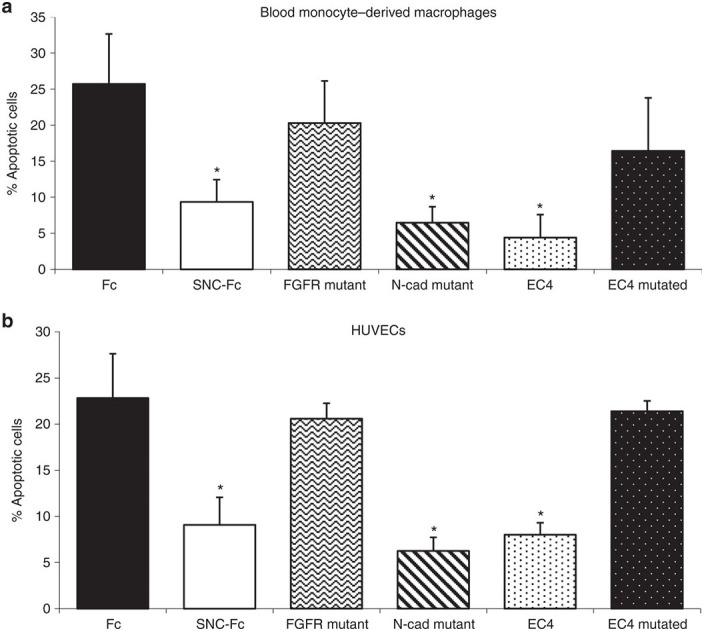
**SNC-Fc and EC4 reduced apoptosis in macrophages and endothelial cells.** Graphs show the percentage of apoptotic (**a**) macrophages and (**b**) endothelial cells as assessed by cleaved caspase-3 immunocytochemistry 24 hours after induction of apoptosis with Fas-L and treatment with the purified proteins (mean ± SEM, *n* = 3, *P < 0.05 versus Fc, fibroblast growth factor receptor (FGFR) mutated and EC4 mutant). EC, extracellular; HUVECs, human umbilical vein endothelial cells; SNC, soluble N-cadherin.

Macrophage and endothelial cell apoptosis are also observed during atherosclerotic plaque progression. EC4-Fc reduced apoptosis of blood monocyte–derived macrophages (Figure 2a) and human umbilical vein endothelial cells (Figure 2b) to an extent similar to that of SNC-Fc. As with VSMCs, this effect was ablated when the FGFR binding site was mutated (Figure 2a,b).

### EC4-Fc reduced atherosclerotic plaque size and increased features of plaque stability

Plasma concentrations of EC4-Fc and SNC-Fc were increased in mice infected with RAd EC4-Fc or RAd SNC-Fc compared with that of RAd Fc–infected control mice. Importantly, there were no significant differences in the plasma levels of low-density lipoprotein of the mice between the groups (Fc: 4.0 ± 1.3 mmol/l, SNC-Fc: 3.9 ± 1.2 mmol/l, EC4-Fc: 4.0 ± 1.4 mmol/l). Additionally, no adverse effects in the mice were observed.

**Figure 3:**
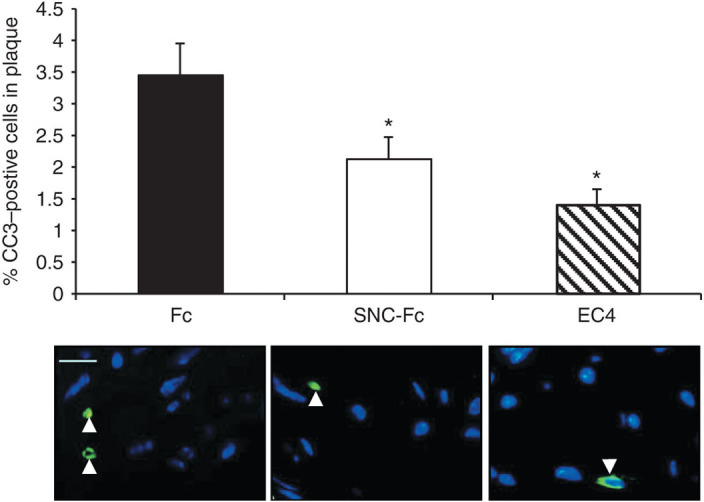
**EC4-Fc reduces apoptosis *in vivo*.** (**a**) Quantification of the percentage of cleaved caspase-3–positive cells within the plaque (mean ± SEM, Fc *n* = 14, SNC *n* = 11, EC4 *n* = 13, *P < 0.05 versus Fc). (**b**) Representative cleaved caspase-3 immunohistochemistry (green) with 4′,6-diamidino-2-phenylindole nuclear staining (blue) of brachiocephalic artery plaques. Bar = 50 µm. EC, extracellular; SNC, soluble N-cadherin.

**Figure 4:**
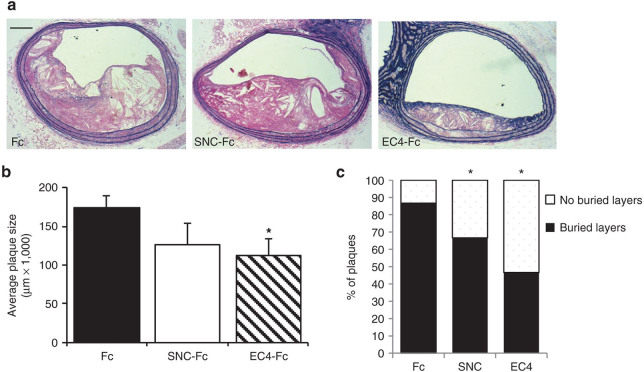
**EC4-Fc reduces atherosclerotic plaque area and incidence of buried layers**. (**a**) Representative elastin van Gieson stained plaques. Bar = 250 µm. (**b**) Analysis of plaque area, an average was taken from four measurements along the brachiocephalic artery. (**c**) Analysis of buried layers. “Unstable” plaques have at least one buried layer, “stable” plaques have none. Mean + SEM, Fc *n* = 15, SNC *n* = 12, EC4 *n* = 15, *P < 0.05 versus Fc). EC, extracellular; SNC, soluble N-cadherin.

Fluorescent immunohistochemistry for cleaved caspase-3 showed that apoptosis within atherosclerotic plaques from the brachiocephalic artery was significantly reduced to a similar extent in the mice infected with RAd EC4-Fc and RAd SNC-Fc (Figure 3). Plaque size was measured at four points along the length of the brachiocephalic artery, and the average was calculated. Interestingly, plaque size was significantly reduced in the mice infected with RAd EC4-Fc compared with that of Fc control mice (Figure 4a,b). No significant effect on plaque size was observed in mice infected with RAd SNC-Fc.

**Figure 5:**
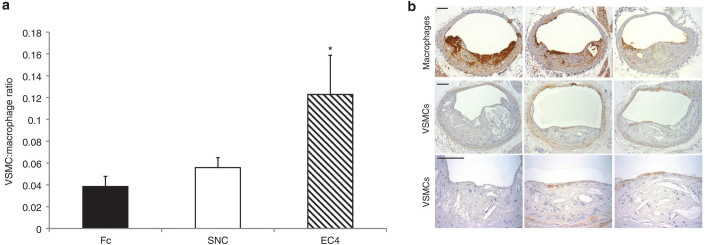
**EC4-Fc increases the ratio of smooth muscle cells to macrophages (a marker of plaque stability)**. (**a**) Analysis of the ratio of vascular smooth muscle cells (VSMCs; actin) to macrophages (Griffonia simplicifolia lectin-1 (GSL); mean ± SEM, Fc *n* = 11, SNC-Fc *n* = 10, EC4-Fc *n* = 10, *P < 0.05 versus Fc). (**b**) Representative images of GSL immunohistochemistry for macrophages and actin immunohistochemistry for VSMCs. Higher power images of the actin immunohistochemistry are shown in lower panels for clarity. Bar = 250 µm. EC, extracellular; SNC, soluble N-cadherin.

The proportion of plaques exhibiting buried fibrous layers (a surrogate marker of plaque instability) were significantly reduced (>35%) in the mice infected with RAd EC4-Fc or RAd SNC-Fc compared with that of the Fc control mice (Figure 4a,c). These data suggest that EC4-Fc has an effect similar to that of SNC-Fc *in vivo* and are in line with our previous findings.^8^ Additionally, we observed a significant increase (approximately threefold) in the ratio of VSMCs:macrophages, using specific cell type markers (Figure 5). This is further confirmation of a more stable plaque phenotype. As observed in our previous study, there were no differences in other plaque parameters measured (Table 1).

## Discussion

VSMC apoptosis is thought to be a major contributor to atherosclerotic plaque instability. Therefore, a therapeutic agent capable of reducing VSMC apoptosis in the fibrous cap of atherosclerotic plaques could be of great clinical benefit. We have previously shown that SNC-Fc significantly reduced apoptosis and increased features of atherosclerotic plaque stability.^8^ However, the therapeutic potential of SNC-Fc is limited due to the size of the molecule (~120 kDa). In the current study, we therefore aimed to identify the active antiapoptotic site within SNC and generate a smaller molecule, which may have greater therapeutic application. We have identified the active site of SNC-Fc as located within the EC4 domain and evaluated the effects of a truncated form of the molecule (~50 kDa and termed EC4-Fc) on apoptosis and atherosclerotic plaque progression. This study clearly identifies the potential for therapeutic strategies targeted at activating FGFR, in reducing the destabilization of atherosclerotic plaques.

SNC-Fc consists of five extracellular domains (EC1–EC5). Motifs within EC1 are responsible for interactions with N-cadherin, and another motif in EC4 is responsible for interactions with the FGFR. To investigate whether these sites are required for the previously observed antiapoptotic effect of SNC-Fc, we mutated the two sites independently of each other or in tandem to prevent their interactions. Interestingly, the antiapoptotic effects of SNC-Fc were modulated through the FGFR binding site within the EC4 domain. We have previously shown that SNC-Fc activates the FGFR.^8^ We therefore postulate that a direct interaction between SNC and FGFR is essential for subsequent prosurvival signaling. In support of these observations, N-cadherin and FGFR have also been shown to interact during neurite outgrowth, as reviewed in ref. 13. The smaller EC4-Fc reduced apoptosis to the same extent as that of SNC-Fc and activated the same prosurvival signaling pathway (Akt phosphorylation). EC4-Fc also reduced endothelial cell and macrophage apoptosis, to a level similar to that of SNC-Fc.

Moreover *in vivo*, elevated plasma levels of EC4-Fc reduced intraplaque frequencies of apoptosis, the number of buried layers, and plaque size when compared with the Fc control. In contrast and in accordance with our previous findings,^8^ elevated levels of SNC-Fc reduced apoptosis and the number of buried layers but had no significant effect on plaque size. Also beneficially for plaque stability, both SNC-Fc and EC4-Fc significantly increased the smooth muscle cell:macrophage ratio. Taken together, these data demonstrate that both SNC and EC4, through interaction with the fibroblast growth factor receptor type 1, can prevent atherosclerotic plaque progression. Although it may appear counter-intuitive that EC4-Fc reduced macrophage apoptosis and the proportion of macrophages in the plaque, we suggest that the lower levels of apoptosis results in less secondary necrosis, inflammation, and proinflammatory cytokine production, leading to reduced influx of macrophages to the plaque.^5,14–17^

Our findings illustrate that in comparison to SNC, EC4 can act as a potent mimetic without any loss in functionality. Indeed, the *in vivo* study suggested that EC4 possesses greater activity as evidenced by the significant reduction in plaque area that was not observed in SNC-Fc animals. We postulate that the smaller molecular weight of EC4 may increase its permeability and therefore augment its access to intraplaque cells. Consequently in future studies, we will aim to design and test small peptides targeting the FGFR binding site. Indeed, a small peptide encoding the N-cadherin binding site in EC1, producing an HAV peptide which antagonises N-cadherin, is being trialed as a cancer chemotherapeutic,^18^ demonstrating that this approach has potential for clinical use.

In conclusion, this study demonstrates that site-specific truncation of SNC (EC4) does not diminish its antiapoptotic activity. The ~50 kDa protein EC4-Fc reduced VSMC, endothelial cell, and macrophage apoptosis *in vitro*, and more importantly, reduced apoptosis, plaque size, and the number of buried layers within murine brachiocephalic artery atherosclerotic plaques, rendering them with an enhanced stable phenotype. Our findings, therefore, suggest that EC4-Fc, or possibly a smaller molecule, may have potential as a therapeutic in the treatment of atherosclerosis.

## Materials and Methods

### VSMC culture

Human saphenous vein VSMCs at passages 4–8 were generated as described.^4^ Ethical permission for this study was gained from the local research ethics committee (NRES 07/H0107/61), and the study was approved by the University of Bristol and National Health Service Review Boards. Each experiment was carried out with VSMCs from at least three different segments of vein.

### Purification and culture of mouse blood monocytes

Mouse peripheral blood monocytes were purified using Ficoll-Hypaque gradient (Ficoll-Paque Plus; Amersham Biosciences, Little Chalfont, UK), followed by differential adherence and culture in 20 ng/ml of macrophage colony-stimulating factor for 7–10 days to induce differentiation into macrophages.

### SNC-Fc mutation and truncation

Two fragments containing the binding sites within EC1 and EC4 were subcloned to facilitate polymerase chain reaction (PCR) mutagenesis. The first contained the HAV and INPISGQ motifs, which are required for interaction with N-cadherin,^9^ and was removed from SNC-Fc using BamHI and HindIII. The second contained the IDPVNGQ motif, which is required for interaction with FGFRs,^9^ and was removed from SNC-Fc using EcoRV and KpnI. These fragments were cloned into the pDRIVE cloning vector (Qiagen, Manchester, UK). PCR mutagenesis was used to mutate the binding sites of SNC. This produced the following mutations in the SNC sequence: HAV → HGV, INPISGQ → INPASGQ, IDPVNGQ → IDAVNGQ, which were validated by sequencing. These mutated fragments were then shuttled back into SNC using the same restriction enzyme sites, to produce two plasmids: “N-cadherin mutated” and “FGFR mutated.” The native, mutated SNC constructs were then cloned into pCpG-free mcs (Invivogen, Toulouse, France) in which a myc-tagged FC domain (FCmyc) had already been cloned. The Fc domain, containing mutations in the immunoglobulin G receptor and complement binding domains, was amplified by PCR from an IL-10-Fc fusion plasmid (generously provided by Terry Storm, Harvard University, Cambridge, MA). This chimeric molecule comprised of SNC and an antibody constant domain, which enables protein A binding, as well as extending plasma half-life^19–21^ and possibly increasing receptor–ligand interaction through its potential for dimerization. Such immunoadhesins have been used *in vivo* as potential therapeutic agents.^22^

The EC4 truncation was produced as follows. To facilitate secretion, the signal prepropeptide of SNC was cloned by PCR creating a BamHI site at the 3′ end into FCmyc. This soluble Fc construct was used as a control (sFCmyc). The EC4 domain of SNC was excised by PCR introducing BamHI sites at each end. This product was cut with BamHI and ligated into sFCmyc to create sEC4-FCmyc and was validated by sequencing.

### Adenovirus production

EcoRI fragments were prepared from sFCmyc or sEC4-FCmyc, which included the mouse cytomegalovirus enhancer, EF1α promoter, a short intron, the coding sequence, and a polyA site, and were cloned into pDC511 (Microbix, Ontario, Canada). Both were recombined with the adenovirus genomic plasmid by cotransfection into 293 cells. The resultant adenoviruses were termed RAd EC4-Fc and RAd Fc (control). RAd SNC-Fc was made as described.^8^

### Purification of Fc, SNC-Fc, and mutated versions of SNC-Fc and EC4-Fc

Chinese hamster ovary cells were infected with 50 pfu/cell of RAd Fc, RAd SNC-Fc, or RAd EC4-Fc or subjected to AMAXA technology–mediated transfection of plasmids for mutated versions of SNC-Fc. The conditioned media was collected at 66 and 138 hours after infection/transfection. The conditioned media was pooled, and protein purification was achieved with protein A columns (Amersham Biosciences). The protein concentration was determined using the Bradford Protein assay (Sigma) and compared using western blotting.

### Induction of VSMC, macrophage, and endothelial cell apoptosis

To enable the detection of apoptotic frequencies by immunocytochemistry, all cells were grown on glass coverslips. Human VSMC apoptosis was induced by culturing in serum-free media with 200 ng/ml of Fas-L for 24 hours. Blood-derived macrophage apoptosis was induced by culture in serum-free media for 72 hours. Human umbilical vein endothelial cell apoptosis was induced by culture in serum-free media for 24 hours. Cells were supplemented with 20 pmol/l of the respective purified proteins to assess their antiapoptotic effects.

### Immunocytochemistry

Apoptosis was assessed by cleaved caspase-3 immunocytochemistry as described previously.^7^ Phosphorylated Akt was detected by immunocytochemistry using rabbit anti-pAkt antibody (Cell Signaling, Danvers, MA), as described previously.^8^

### *In vivo* mouse experiments

Homozygous C57BL/6, 129 male apolipoprotein E^−/−^ mice (strain background 71% C57BL/6 and 29% 129) were bred within the Animal Unit of the University of Bristol. Housing, care, and all procedures were performed in accordance with the guidelines and regulations of the University of Bristol and the United Kingdom Home Office, and this study was approved by the University of Bristol Review Board. To induce the formation of complex atherosclerotic lesions, 8-week-old male apolipoprotein E^−/−^ mice were fed a high-fat rodent diet containing 21% (w/w) fat from lard supplemented with 0.15% (w/w) cholesterol (Special Diets Services, Witham, UK) for a period of 8 weeks. Mice were then given an i.v. dose of 8 × 10^10^ viral particles of an empty adenovirus (RAd66) as a predose 4 hours before administration of 2.25 × 10^8^ viral particles of RAd SNC-Fc (*n* = 12), RAd EC4-Fc (*n* = 14), or Fc (*n* = 15) as previously described.^23^ This protocol allows efficient hepatic transfection and expression, and subsequent elevated circulating levels of the respective transgene. Mice were then maintained on a high-fat diet for a further 28 days to determine effects on established lesions.

### Quantification of plasma SNC-Fc/EC4 and lipoprotein levels

Plasma samples were taken at 6 and 28 days after RAd administration, and the levels of SNC-Fc and EC4 were analyzed using western blotting. For the SNC-Fc blots, plasmas were pretreated with protein A beads for 30 minutes at room temperature. Beads were centrifuged for 5 minutes at 13,000 rpm, the supernatant was removed, and the beads were resuspended in 30 µl of sodium dodecyl sulfate lysis buffer. These samples were subjected to western blotting as described previously.^4^ Blots were detected with a goat anti–mouse horseradish peroxidase secondary antibody diluted 1:1,000 in milk overnight. To detect EC4, immunoprecipitation of c-myc using the ProFound c-myc tag IP/co-IP kit (Fisher Scientific, Loughborough, UK) was performed. Blots were detected with c-myc tag antibody (Cell Signaling) and a swine anti-rabbit secondary antibody (Dako, High Wycombe, UK).

Plasma lipid profiles were analyzed in terminal plasma samples as previously described.^24^

### Immunohistochemistry

VSMCs, macrophages, and proliferating and apoptotic cells were identified by immunohistochemistry for α-smooth muscle cell actin, Griffonia simplicifolia lectin-1, proliferating cell nuclear antigen, and cleaved caspase-3 as described previously.^8,23^ Collagen was identified by PicroSirius red staining observed under polarized light.^8,23^ Positive staining was quantified using a computerized image analysis system (Image Pro Plus; Media Cybernetics, Rockville, MD) and expressed as the percentage of total plaque area.

### Identification of buried fibrous layers

Serial sections stained for elastin and α-smooth muscle cell actin were examined for the presence of structures rich in elastin and VSMCs, and these were classed as buried fibrous layers, which we have previously identified as a surrogate marker of previous plaque instability, as previously described.^25^

### Statistical analysis

Values are expressed as mean ± SEM. Data were tested for normality. Then normally distributed data were analyzed by analysis of variance for multiple comparisons and the Student–Newman–Keuls posttest, and for experiments with two groups, the paired *t*-test was utilized. Differences were considered significant when *P* < 0.05.

## Figures and Tables

**Table 1:** The effect of EC4-Fc on atherosclerotic plaques in the mouse brachiocephalic artery

	Fc (*n* = 14)	SNC-Fc (*n* = 11)	EC4-Fc (*n* = 13)
Cleaved caspase-3 (% total cells)	3.5 ± 0.5	2.1 ± 0.4^*^	1.4 ± 0.2^*^
VSMCs (% total plaque area)	0.8 ± 0.2	2.4 ± 0.6^*^	1.8 ± 0.4^*^
Fibrous cap coverage (% total plaque area)	12.2 ± 3.3	18.2 ± 4.4	20.3 ± 4.8
Fibrous cap thickness (average)	65.03 ± 6.7	56.38 ± 8.8	67.8 ± 5.3
Fibrous cap thickness (minimum)	10.1 ± 1.0	11.6 ± 2.7	10.6 ± 1.5
Macrophages (% total plaque area)	23.6.0 ± 3.8	21.6 ± 3.0	14.5 ± 2.1^*^
VSMC: macrophage ratio	0.038 ± 0.009	0.056 ± 0.009	0.122 ± 0.036^*^
Collagen (% of total plaque area)			
Polarized	5.69 ± 0.9	4.90 ± 1.0	5.07 ± 1.0
Brightfield	50.15 ± 2.4	51.14 ± 3.1	52.66 ± 2.3
Lipid content (percentage of total plaque area)	51.09 ± 2.4	49.93 ± 3.1	49.35 ± 2.4
Necrotic core (percentage of total plaque area)	35.17 ± 4.0	54.38 ± 17.3	23.93 ± 5.3

Values are presented as mean ± SEM.

* Indicates significant difference from Fc, analysis of avriance, and Student–Newman–Keuls posttest .

SNC, soluble N-cadherin; VSMC, vascular smooth muscle cell.

## References

[bib1] Bauriedel G Hutter R Welsch U Bach R Sievert H Lüderitz B 1999 Role of smooth muscle cell death in advanced coronary primary lesions: implications for plaque instability Cardiovasc Res 41 480 488 10341848 10.1016/s0008-6363(98)00318-6

[bib2] Geng YJ Libby P 1995 Evidence for apoptosis in advanced human atheroma. Colocalization with interleukin-1 beta-converting enzyme Am J Pathol 147 251 266 7639325 PMC1869820

[bib3] Dhume AS Soundararajan K Hunter WJ 3rd Agrawal DK 2003 Comparison of vascular smooth muscle cell apoptosis and fibrous cap morphology in symptomatic and asymptomatic carotid artery disease Ann Vasc Surg 17 1 8 12522697 10.1007/s10016-001-0331-1

[bib4] Uglow EB Slater S Sala-Newby GB Aguilera-Garcia CM Angelini GD Newby AC 2003 Dismantling of cadherin-mediated cell-cell contacts modulates smooth muscle cell proliferation Circ Res 92 1314 1321 12775583 10.1161/01.RES.0000079027.44309.53

[bib5] Clarke MC Figg N Maguire JJ Davenport AP Goddard M Littlewood TD 2006 Apoptosis of vascular smooth muscle cells induces features of plaque vulnerability in atherosclerosis Nat Med 12 1075 1080 16892061 10.1038/nm1459

[bib6] Zadelaar AS von der Thüsen JH Boesten LS Hoeben RC Kockx MM Versnel MA 2005 Increased vulnerability of pre-existing atherosclerosis in ApoE-deficient mice following adenovirus-mediated Fas ligand gene transfer Atherosclerosis 183 244 250 15927188 10.1016/j.atherosclerosis.2005.03.044

[bib7] Koutsouki E Beeching CA Slater SC Blaschuk OW Sala-Newby GB George SJ 2005 N-cadherin-dependent cell-cell contacts promote human saphenous vein smooth muscle cell survival Arterioscler Thromb Vasc Biol 25 982 988 15774907 10.1161/01.ATV.0000163183.27658.4b

[bib8] Lyon CA Johnson JL Williams H Sala-Newby GB George SJ 2009 Soluble N-cadherin overexpression reduces features of atherosclerotic plaque instability Arterioscler Thromb Vasc Biol 29 195 201 19008530 10.1161/ATVBAHA.108.178087 PMC2853707

[bib9] Doherty P Walsh FS 1996 CAM-FGF Receptor Interactions: A Model for Axonal Growth Mol Cell Neurosci 8 99 111 10.1006/mcne.1996.0049 8954625

[bib10] Li G Satyamoorthy K Herlyn M 2001 N-cadherin-mediated intercellular interactions promote survival and migration of melanoma cells Cancer Res 61 3819 3825 11325858

[bib11] Rieger-Christ KM Lee P Zagha R Kosakowski M Moinzadeh A Stoffel J 2004 Novel expression of N-cadherin elicits *in vitro* bladder cell invasion via the Akt signaling pathway Oncogene 23 4745 4753 15122336 10.1038/sj.onc.1207629

[bib12] Tran N Adams D Vaillancourt R Heimark R 2002 Signal transduction from N-cadherin increases Bcl-2 J Biol Chem 277 32905 32914 12095980 10.1074/jbc.M200300200

[bib13] Williams EJ Williams G Howell FV Skaper SD Walsh FS Doherty P 2001 Identification of an N-cadherin motif that can interact with the fibroblast growth factor receptor and is required for axonal growth J Biol Chem 276 43879 43886 11571292 10.1074/jbc.M105876200

[bib14] Schaub FJ Han DK Liles WC Adams LD Coats SA Ramachandran RK 2000 Fas/FADD-mediated activation of a specific program of inflammatory gene expression in vascular smooth muscle cells Nat Med 6 790 796 10888928 10.1038/77521

[bib15] Schaub FJ Liles WC Ferri N Sayson K Seifert RA Bowen-Pope DF 2003 Fas and Fas-associated death domain protein regulate monocyte chemoattractant protein-1 expression by human smooth muscle cells through caspase- and calpain-dependent release of interleukin-1alpha Circ Res 93 515 522 12946945 10.1161/01.RES.0000093205.42313.7C

[bib16] Khan M Pelengaris S Cooper M Smith C Evan G Betteridge J 2003 Oxidised lipoproteins may promote inflammation through the selective delay of engulfment but not binding of apoptotic cells by macrophages Atherosclerosis 171 21 29 14642402 10.1016/j.atherosclerosis.2003.07.001

[bib17] Grainger DJ Reckless J McKilligin E 2004 Apolipoprotein E modulates clearance of apoptotic bodies *in vitro* and in vivo, resulting in a systemic proinflammatory state in apolipoprotein E-deficient mice J Immunol 173 6366 6375 15528376 10.4049/jimmunol.173.10.6366

[bib18] Burden-Gulley SM Gates TJ Craig SE Lou SF Oblander SA Howell S 2009 Novel peptide mimetic small molecules of the HAV motif in N-cadherin inhibit N-cadherin-mediated neurite outgrowth and cell adhesion Peptides 30 2380 2387 19765627 10.1016/j.peptides.2009.09.013

[bib19] Capon DJ Chamow SM Mordenti J Marsters SA Gregory T Mitsuya H 1989 Designing CD4 immunoadhesins for AIDS therapy Nature 337 525 531 2536900 10.1038/337525a0

[bib20] Zheng XX Steele AW Nickerson PW Steurer W Steiger J Strom TB 1995 Administration of noncytolytic IL-10/Fc in murine models of lipopolysaccharide-induced septic shock and allogeneic islet transplantation J Immunol 154 5590 5600 7730658

[bib21] Barrou B Bertry-Coussot L Morin S Sainz J Lucas B Bitker MO 2002 Prolonged islet allograft survival by adenovirus-mediated transfer of sICAM-1/Ig immunoadhesin gene Hum Gene Ther 13 1441 1450 12215265 10.1089/10430340260185076

[bib22] Chamow SM Ashkenazi A 1996 Immunoadhesins: principles and applications Trends Biotechnol 14 52 60 8746117 10.1016/0167-7799(96)80921-8

[bib23] Johnson JL Baker AH Oka K Chan L Newby AC Jackson CL 2006 Suppression of atherosclerotic plaque progression and instability by tissue inhibitor of metalloproteinase-2: involvement of macrophage migration and apoptosis Circulation 113 2435 2444 16702468 10.1161/CIRCULATIONAHA.106.613281

[bib24] Johnson JL Jackson CL 2001 Atherosclerotic plaque rupture in the apolipoprotein E knockout mouse Atherosclerosis 154 399 406 11166772 10.1016/s0021-9150(00)00515-3

[bib25] Johnson J Carson K Williams H Karanam S Newby A Angelini G 2005 Plaque rupture after short periods of fat feeding in the apolipoprotein E-knockout mouse: model characterization and effects of pravastatin treatment Circulation 111 1422 1430 15781753 10.1161/01.CIR.0000158435.98035.8D

